# Emerging Technologies to Create Inducible and Genetically Defined Porcine Cancer Models

**DOI:** 10.3389/fgene.2016.00028

**Published:** 2016-02-29

**Authors:** Lawrence B. Schook, Laurie Rund, Karine R. Begnini, Mariana H. Remião, Fabiana K. Seixas, Tiago Collares

**Affiliations:** ^1^University of Illinois Cancer Center, University of Illinois at ChicagoChicago, IL, USA; ^2^Department of Animal Sciences, University of Illinois at Urbana–ChampaignChampaign, IL, USA; ^3^Postgraduate Program in Biotechnology, Biotechnology Unit, Technology Development Center, Federal University of PelotasPelotas, Brazil

**Keywords:** oncopigs, cancer, biotechnology, transgenesis, genome editing

## Abstract

There is an emerging need for new animal models that address unmet translational cancer research requirements. Transgenic porcine models provide an exceptional opportunity due to their genetic, anatomic, and physiological similarities with humans. Due to recent advances in the sequencing of domestic animal genomes and the development of new organism cloning technologies, it is now very feasible to utilize pigs as a malleable species, with similar anatomic and physiological features with humans, in which to develop cancer models. In this review, we discuss genetic modification technologies successfully used to produce porcine biomedical models, in particular the Cre-*loxP* System as well as major advances and perspectives the CRISPR/Cas9 System. Recent advancements in porcine tumor modeling and genome editing will bring porcine models to the forefront of translational cancer research.

## Introduction

Animal models have played a central role over the centuries in scientific investigations of human disease and treatment strategies. Genetic strategies for the development of cancer models using human mutations in targeted oncogenic pathways demonstrated that porcine fibroblasts could be transformed *in vitro* and could be tumorigenic with four to six gene alterations (Adam et al., 2007). The authors used retroviral vectors carrying pairs of human and murine oncogenic cDNAs (hTERT and p53^DD^, cyclin D1 and CDK4^R24C^, and c-Myc^T58A^, and H-Ras^G12V^) to transform porcine fibroblasts. These altered cells showed a transformed phenotype in culture and formed tumors following autologous transfer. These induced changes demonstrated that the pig/tumorigenic pathway recapitulated those observed in human much more closely than murine cells (Adam et al., 2007). Although this approach was limited because the animals needed to be immuno-suppressed for tumors to grow *in vivo*, this work was the first to demonstrate that genetically defined tumors could be induced in a large animal (Schook et al., 2015a).

Recent innovations in reproductive, cloning and transgene technologies have enhanced efficacy and efficiency or producing targeted porcine genome modifications. With the successful cloning of animals by somatic cell nuclear transfer (SCNT), it is now possible to produce genetically modified pigs from genetically engineered somatic donor cells using a wide variety of techniques from random genomic insertion of plasmid DNA (Hyun et al., 2003; Watanabe et al., 2005), to genomic integration of transduced retroviral or lentiviral vectors (Lai et al., 2002; Park et al., 2002), and to modern genome editing with molecular methods using endonucleases such as transposases, recombinases, and programmable nucleases (Zhou et al., 2015). Genetic modification technologies successfully used to produce porcine biomedical models, in particular the Cre-*loxP* System as well as major advances and perspectives the CRISPR/Cas9 System will be presented in this mini review.

## Cre-*loxP* System

The ability to activate latent genes in defined tissues and at defined times is a key factor to enable the development of inducible temporally and spatially regulated cancer models. The activation of an oncogenic mutation(s) in a chosen tissue could mimic the spontaneous somatic events that initiate many human cancers and enable replication of diverse cancer types using the same mutant gene(s) (Flisikowska et al., 2013). Those conditional gene expressions are now well established in mice using site-specific recombinase (SSR) systems that allow the precise recombination between genomic sites, resulting in deletion or inversion of the intervening sequences (Frese and Tuveson, 2007; Oh-McGinnis et al., 2010). The use of SSR technology in genome manipulation has been demonstrated to effectively resolve complex transgene insertions to single copy, remove unwanted DNA, and precisely insert DNA into known genomic target sites (Wang et al., 2011). Site-specific recombination occurs at a specific sequence or recognition site and involves cleavage and reunion leading to integration, deletion or inversion of a DNA fragment without the gain or loss of nucleotides (Wang et al., 2011). Because of the efficiency of the SSR systems, it can be applied to conditional deletions of relatively short coding sequences or regulatory elements but also to more extensive chromosomal rearrangement strategies (Oh-McGinnis et al., 2010).

Cre-recombinase system is one of the best-studied and most commonly used SSR in mammalian cell cultures. Since its first use for mammalian genome editing in 1988 (Sauer and Henderson, 1988) many adaptations have expanded the utility of the Cre system from flies to mammalian cells beyond mouse to include porcine and humans cell lines (Lanza et al., 2012). Cre-recombinase is derived from the bacteriophage P1 and recognizes a distinct sequence-specific motif termed as recombination target sites (*loxP*) catalyzing efficient conservative DNA rearrangements (Wirth et al., 2007). The *loxP* site is a 34 bp palindromic sequence with an 8-bp asymmetric spacer region (Feng et al., 1999; Siegel et al., 2001; Araki et al., 2002; Sauer, 2002; Schnutgen et al., 2003; Garcia-Otin and Guillou, 2006) and acts upon the neighboring DNA sequences. The Cre-*loxP* system is a bidirectional tyrosine recombinase that enables the recombinase-mediated genetic cross-over between two identical *loxP* recognition sites promoting intermolecular or intramolecular recombination. Intermolecular recombination is a translocation between two DNA fragments with corresponding *loxP* sites, while the intramolecular recombination involves removal of genetic material between two *loxP* sites, with the last one been the preferred function of Cre-recombinase (Feng et al., 1999). Because of the identical nature of the recognition sites, the recombination reaction is fully reversible, although intramolecular recombination (excision) is highly favored over intermolecular reactions (integration) (Wang et al., 2011).

One of the most powerful and widely used applications of the Cre/*loxP* system is in conditional gene expression (Gu et al., 1994). This strategy allows for tissue and time-specific gene expression when recombination is triggered by Cre-recombinase, and is even more important in cancer models where oncogenic activation in a chosen tissue could mimic the spontaneous somatic events that initiate many human cancers (Schook et al., 2015a). Endogenous engineered mice are usually conditional alleles constructed by the insertion of a transcriptional and translational LoxStopLox ‘stop’ cassette between the promoter and first coding exon of the oncogenic allele. Providing the expression of an active Cre-recombinase, the stop cassette is excised and the mutant oncogene is subsequently expressed (de Alboran et al., 2001; Jackson et al., 2001). In pigs, this conditional gene expression strategy has been used to promote oncogenic expression in three cancer models (Leuchs et al., 2012; Li et al., 2015; Schook et al., 2015b). Leuchs et al. (2012) have generated gene-targeted pigs with a conditionally activated oncogenic mutant form of p53, which in latent form is a gene knockout. The construction used a porcine BAC vector with CAGGS promoter-mCherry cassette (in reverse orientation) as a fluorescent counter-selectable marker; a short arm of homology corresponding to a region of TP53 intron 1 from a point of exon 2 to a *PmlI* restriction enzyme site of exon 2; a floxed transcriptional termination cassette (LSL); and a region extending from the *PmlI* site in intron 1 to a point of exon 11 that includes a G to A substitution in exon 5 changing arginine to histidine in codon 167 (R167H) (Leuchs et al., 2012). In this same model, viable gene-targeted pigs carrying a latent *Kras*^G12D^ mutant allele that could be activated by Cre-recombinase was constructed (Li et al., 2015). The KRAS-neo vector comprised: a short homology arm in *KRAS* intron 1; a transcriptional stop cassette comprising: a *loxP* site; adenoviral splice acceptor; promoterless neomycin phosphotransferase resistance gene (*neo*); three poly-adenylation signals derived from SV40, bovine growth hormone and cytomegalovirus; and a second *loxP* site inserted into a *ClaI* site in KRAS intron 1; and a region of porcine KRAS extending from the *ClaI* site in intron 1 to a *SacI* site in intron 2, which also included an engineered G to A point mutation within exon 2 that results in a glycine to aspartic acid substitution at codon 12 (G12D) (Li et al., 2015). Both *KRAS* and *TP53* transgenic pigs cells were transduced with 5 μM of Cre protein produced *in vitro* with the vector pTriEx-HTNC (Addgene plasmid 13763; Leuchs et al., 2012; Li et al., 2015).

**FIGURE 1:**
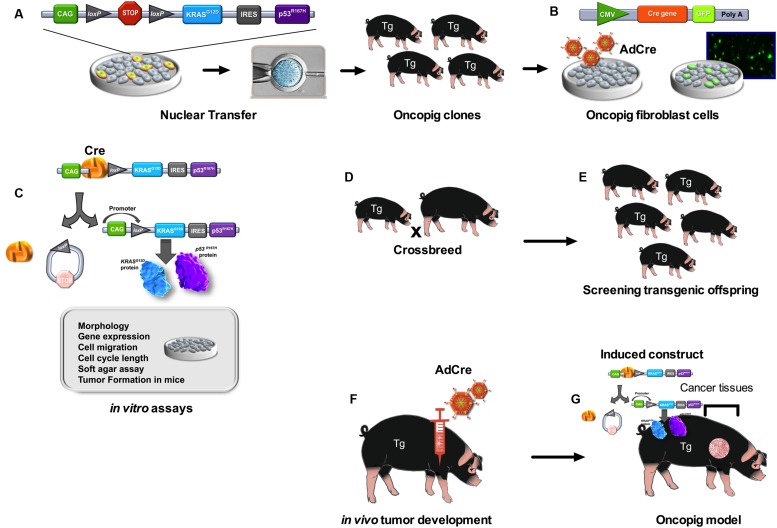
**Development of the Oncopig using the Cre-*loxP* inducible system.** Pigs were cloned from fibroblast cells with this transgene construct **(A)**. Fibroblast cell lines were established from oncopig offspring. *In vitro* work utilized fibroblast cell lines isolated from transgenic oncopigs which were then infected with adenovirus encoding Cre recombinase (AdCre) and the marker green fluorescent protein (GFP) **(B)**. AdCre induced removal of the STOP codon allowing for expression of both transgenes. This expression altered the phenotype of the cells as was demonstrated in numerous *in vitro* assays **(C)**. Oncopig clones (founders) were crossbreed with York females **(D)**. The transgenic offspring (Tg) **(E)** were injected with AdCre at various sites including intramuscular, subcutaneous and intratesticular sites **(F)**. Tumors were induced at each site of injection in transgenic oncopigs **(G)**.

Transgenic oncopigs (**Figure 1**) have also been engineered to contain oncogenic *Kras*^G12D^ and dominant-negative p53^R167H^ downstream of a LoxP-polyA(STOP)-LoxP sequence (LSL) and CAG promoter (Schook et al., 2015b). Site-directed mutagenesis was then used to introduce the oncogenic G12D mutation into the porcine *KRAS* cDNA and the R167H mutation was chosen for *TP53* as its human equivalent (R175H) is commonly found in human cancers as well as the cancer predisposition Li-Fraumeni Syndrome. These two cDNAs were then introduced into a Cre-inducible vector, followed by the aforementioned LSL sequence, *KRAS*^G12D^, an IRES sequence to allow for bicistronic expression, *TP53*^R167H^ and a poly A sequence. This design allows for co-expression of both *KRAS*^G12D^ and *TP53*^R167H^ in ostensibly any cells of the pig by transient expression of AdCre (Ad5CMVCre-eGFP, AdGFP, Gene Transfer Vector Core; Schook et al., 2015b). These pig models have resulted in tumorigenic profiles *in vitro* (Leuchs et al., 2012; Li et al., 2015) and *in vivo* (Schook et al., 2015b) and the results obtained with these three cancer pig models are shown in **Table 1**.

**Table 1:** Porcine models for cancer developed by different strategies.

Author	Gene	Technique	Inducible strategy	Survival	Location	Phenotype	Tumor progression
Yamakawa et al., 1999	*v-Ha-ras*	Pronuclear microinjection	No	_	_	No	No
McCalla-Martin et al., 2010	Gli2	DNA vector + SCNT	No	Euthanized due to bacterial infection	_	Not informed	Not informed
Luo et al., 2011	BRCA-1 (KO)	Adenovirus + SNCT and HMC	No	18 days	Breast	Not informed	Not informed
Flisikowska et al., 2013	*APC*	DNA vector + SCNT	No	At least 1 year	Intestinal polyps	Yes	Yes
Leuchs et al., 2012	*TP53*	DNA vector + SNCT	Cre-recombination	Not informed	*_*	Yes	Not yet
Sieren et al., 2014	*TP53*	rAAV + SCNT	No	Not informed	Several locations	Yes	Yes
Li et al., 2015	*Kras*	DNA vector + SNCT	Cre-recombination	Yes	_	Not informed	Not informed
Schook et al., 2015b	*TP53 + Kras*	DNA vector + SNCT	Cre-recombination	Yes	Several locations	Yes	Yes

*SCNT, Somatic Cell Nuclear Transfer; HMC, Handmade Cloning.*

## CRISPR/Cas9 System

The discovering of molecules that recognize specific sequences of DNA was one of the most important advances in gene editing technology allowing site specific genetic modifications to be made. These DNA binding proteins include the zinc fingers and transcriptional activator-like effector (TALE; Wood et al., 2011; Gaj et al., 2013). When they are fused to nucleases, they generate a double-strand break (DSB) in the DNA at the desired genomic loci, triggering the endogenous DNA repair machinery (Gaj et al., 2013; Zhu et al., 2014); if fused to transcription factors or inhibitor molecules, they can bind to promoter regions of target genes, modulating gene expression (Gilbert et al., 2014; Kearns et al., 2014). However, there is a disadvantage of utilizing these proteins that interact with DNA: production of these proteins involves a complicated and more expensive assembly process (Pan et al., 2014).

An easier, cheaper, and yet highly efficient tool for directed genome edition appeared to be more worthwhile and profitable than proteins: the CRISPR (clustered regularly interspaced short palindromic repeat)/Cas (CRISPR associated proteins) system. This system is simpler then zinc fingers and TALEs because the CRISPR/Cas system uses the RNA-DNA interaction for genome loci recognition, which is more specific than protein-DNA (Gasiunas and Siksnys, 2013; Pan et al., 2014).

CRISPR/Cas system has been recently discovered as an adaptive immune system of some bacteria and archaea and protects them against invading viruses and plasmids (Barrangou et al., 2007). The transcription of the repeat-spacer elements from CRISPR locus generates a precursor non-coding CRISPR RNA (pre-crRNA) that later will be cleaved to have short CRISPR RNAs (crRNA) (Garneau et al., 2010; Jinek et al., 2012). The crRNA will be homologous to the DNA or RNA from foreign sequences, and when the invasion occurs, the crRNA will be directed just by Watson-Crick base pairing (Jinek et al., 2012; Wade, 2015). There are different types of CRISPR systems in different organisms (I–III), and the one that have been most developed as a new tool for genome editing, the CRISPR/Cas9 system, is the type II CRISPR originating from *Streptococcus pyogenes* SF370 (Jinek et al., 2012; Qi et al., 2013). The type II is different from types I and III, that crRNA hybridize with another RNA molecule, the *trans*-activating crRNA (tracrRNAs), to direct Cas9 protein to specific DNA sequences (Jinek et al., 2012; Mali et al., 2013; Doudna and Charpentier, 2014). For genome editing, the researchers created a single chimeric guide RNA’s (sgRNA), which is a fusion of a precursor crRNA and a transactivating crRNA (tracrRNA) (Jinek et al., 2012; Pan et al., 2014). Beyond the polymerization, the genome sequence from invader has a complementary genome sequence containing a tri-nucleotide protospacer adjacent motif (PAM) that will be required for initial binding of Cas9 protein (Guilinger et al., 2014). Cas9 protein has an endonuclease activity that cleaves on both strands a few nucleotides away from the PAM generating DSB, preventing the invader genome translation (Jinek et al., 2012). This has been used to generate knockin and knockout transgenic animals, as the DSB activates the endogenous DNA repair machinery by non-homologous joining (Ma et al., 2014; Flemr and Buhler, 2015; Yang, 2015; Zhu et al., 2015).

However, the study of CRISPR/Cas9 identified a new application for Cas9: without its nuclease activity, Cas9 protein, attached to a molecule that modulates gene expression, could bind to the promoter region of some gene of interest, changing the genic expression pattern (Qi et al., 2013). The catalytically dead Cas9 (dCas9), lacking endonuclease activity, contains two mutations in the nuclease domains (D10A and H840A) (Choudhary et al., 2015). Since dCas9 was reported, new studies have been described using it for genome regulation creating different segments to use this tool: CRISPRi, for gene interference, and CRISPRa, for activation of gene translation. When these strategies uses an effector domain attached to dCas9, it can be called CRISPRe. For gene interference (CRISPRi), dCas9 recognizes sgRNA attached to the promoter region of target gene, impairing transcription (Qi et al., 2013). However, this strategy is not efficient for gene repression in eukaryotic cells, so dCas9 can be fused to a transcription repression domain to enhance gene knockdown (Gilbert et al., 2013). The most described strategy for CRISPRi is dCas9 fused to a KRAB (Krüppel- associated box domain of Kox1), a repressive chromatin modifier domain, which have been demonstrating increased gene expression repression in relation to dCas9 alone (Gilbert et al., 2013, 2014). Some authors mention that CRISPRi can be an alternative strategy to RNAi for repressing gene expression in mammalian cells (Gilbert et al., 2013).

Another approach for using dCas9 is fused to transcriptional activator domains, which can be called CRISPRa (Gilbert et al., 2014) or CRISPR-on system (Cheng et al., 2013a), to induce expression of target genes. To achieve that, dCas9 fused to the transcriptional activator is guided by the sgRNA complementary to the promoter region of the gene. The well-characterized tetramer of herpes simplex virus protein, VP16 (VP64) is one of the most reported transcription activator attached to dCas9 and it has been shown to induce gene expression in eukaryotic cells, including human cells (Gilbert et al., 2013, 2014; Maeder et al., 2013; Perez-Pinera et al., 2013; Kearns et al., 2014). Some studies also report that target genes can be simultaneity artificially activated by just adding complementary sgRNAs of promoters of each one of the interest genes (Cheng et al., 2013b; Maeder et al., 2013). This strategy has been tested in human and mouse transformed cells, as well as in ES cells, in one-cell embryo (Cheng et al., 2013b).

The use of CRISPR/Cas9 strategy to build an animal for model of cancer disease is a recently developed approach. For lung adenocarcinoma, Maddalo et al. (2014) describe a methodology of *in vivo* chromosomal rearrangement using CRISPR/Cas9 delivered by virus infection. Rearranging chromosomes by fusing EML4 and ALK genes generated a new murine model for lung adenocarcinoma. An *in vivo* somatic cancer mutation in adult animals was described by Xue et al. (2014), which they developed a different strategy using a hydrodynamic delivery of plasmids with CRISPR components that occasioned to efficient hepatocyte transfection to edit oncogenes and suppressor-tumor genes.

Most frequently, rodents are used to test new strategies for genome editing with CRISPR/Cas9 system to develop cancer and other biomedical models of human disease. However, a new strategy for enrichment of cells with chromosomal deletions made by CRISPR/Cas9 to generate cancer genotype was developed in porcine embryonic fibroblasts (He et al., 2015). For employment in xenotransplants, CRISPR/Cas9 technology has already been applied to inactivate porcine endogenous retroviruses in porcine kidney epithelial cell line (Yang et al., 2015).

Not only modifications in genome sequence can induce cancer phenotype, epigenetic modifications can also be a target to develop animal models for cancer. Falahi et al. (2015) supposes that dCas9 can contribute for epigenome engineering to develop animals for cancer study. Effector domains attached to dCas9 could generate epigenetic mutations known to evolve to different cancer types. Also using dCas9, attached or not to KRAB domain, initial studies in human cells HEK293 and HEK293T, showed repression of TP53 (Lawhorn et al., 2014).

The recent advances generated by CRISPR/Cas9 system in genome editing are extremely important for development of new strategies to generate animal models of cancer. The simplicity, low cost, and low off-target effects put this strategy as one alternative not only for ZFN and TALEN, but also for RNAi technology and Cre-*loxP* systems.

## Perspectives

To unite Cre-*loxP* and CRISPR/Cas9 system has been a promising approach to develop animal models for cancer. Cre-*loxP* affords to conditional gene expression, while CRISPR/Cas9 can be used for target gene insertion and also for gene expression regulation. Some promising works already showed how these technologies can be used together. Using Cre-*loxP* system for induced expression, Sánchez-Rivera et al. (2014) used a system with CRISPR/Cas9 and Cre recombinase to evaluate new candidates for cancer genome, developing adenocarcinoma by editing tumor-suppressor genes sequences in mice models. A different association of both techniques is a study that a mouse model had Cas9 expressed by Cre dependence, and when expressed in conjunction with sgRNAs for Kras, p53, and LKB1 genes, it generated a change of function of those proteins, taking to macroscopic tumors of adenocarcinoma pathology (Platt et al., 2014). Probably, the next step is to standardize those techniques and employ them for a next-generation models for human cancer (Sanchez-Rivera and Jacks, 2015), and pigs fits for those purpose.

## Author Contributions

TC: acquisition of data, data analysis/interpretation, drafting of the manuscript and figure; FS: acquisition of data, data analysis/interpretation, drafting of the manuscript; KB: acquisition of data, data analysis/interpretation, drafting of the manuscript and table; MR: acquisition of data, data analysis/interpretation, drafting of the manuscript; LR: critical revision of the manuscript, drafting of the manuscript and figure; LS: critical revision of the manuscript. All authors approved the manuscript.

## Conflict of Interest Statement

The authors declare that the research was conducted in the absence of any commercial or financial relationships that could be construed as a potential conflict of interest. The reviewer CS and handling Editor declared their shared affiliation, and the handling Editor states that the process nevertheless met the standards of a fair and objective review.
